# Thermodynamically consistent derivation of chemical potential of a battery solid particle from the regular solution theory applied to LiFePO_4_

**DOI:** 10.1038/s41598-019-38635-2

**Published:** 2019-02-14

**Authors:** Klemen Zelič, Tomaž Katrašnik

**Affiliations:** University of Ljubljana, Faculty of Mechanical Engineering, Laboratory for Internal Combustion Engines and Electromobility, Ljubljana, SI-1000 Slovenia

## Abstract

The chemical potential of lithium in *Li*_*x*_*FePO*_4_ active cathode nanoparticles and the surface free energy between *Li*_*x*_*FePO*_4_ and electrolyte were determined with the novel thermodynamically consistent application of the regular solution theory. Innovative consideration of crystal anisotropy accounts for the consistent determination of the dependency of the chemical potential on the mechanistically derived enthalpy of mixing and the phase boundary gradient penalty. This enabled the analytic, thermodynamically consistent determination of the phase boundary thickness between *LiFe*_*P*_*O*_4_ and *FePO*_4_, which is in good agreement with experimental observations. The obtained explicit functional dependency of the surface free energy on the lithium concentration enables adequate simulation of the initiation of the phase transition from *FePO*_4_ to *LiFePO*_4_ at the surface of active cathode particles. To validate the plausibility of the newly developed approaches, lithium intercalation into the *Li*_*x*_*FePO*_4_ nanoparticles from electrolyte was modeled by solving the Cahn-Hilliard equation in a quasi-two-dimensional domain.

## Introduction

A rapid increase in mobile applications has resulted in intense research on energy storage materials. Due to their large capacity, Li-ion batteries with several possible active cathode materials are very popular. Maximizing the energy and power densities of such materials while ensuring a long cycle life inherently calls for an enhanced understanding of underlying physicochemical processes. This objective recently resulted in intensified research in the area of modeling of lithium intercalation into active cathode material from electrolyte. Phase field models of Li intercalation are based on solving the continuity equation with the chemical potential derived from the regular solution theory. Due to its phase separation nature^1–5^, *Li*_*x*_*FePO*_4_ (LFP) material is among the most challenging, and it is also one of the most extensively modeled cathode materials. The newly developed theory proposed in this paper was therefore derived for and demonstrated on the phase separating LFP material.

Selected state-of-the-art models^2,4,6–15^ offer profound insights into the intercalation process of the LFP, whereas these models feature certain deficiencies related to consideration of the crystal lattice anisotropy and description of the surface-related phenomena at the interface of the cathode material and the electrolyte. Models of the intercalation of lithium into the active cathode material are based on the regular solution theory^16^, which considers intercalated lithium atoms and vacant lithium intercalation sites in the crystal lattice of the cathode material as two diffusing species in the solid solution^17–19^. Application of the regular solution theory to such a system is performed by the attributing the solute function to lithium atoms and vacancies and considering the crystal lattice of the cathode material as solvent^16,18,19^. Originally, regular solution theory was designed for the description of liquid solutions with amorphous material structure^1,16,20–22^. This theory assumes an isotropic nature of the considered media, which is not the case for the crystalline cathode material. The obtained chemical potential is thus independent of the crystallographic properties. This deficiency is usually dealt with in such a way that anisotropy of the crystal lattice is introduced into the model by empirical assignation of a tensor nature to the diffusion coefficient and governing parameters of the chemical potential^6,8^. Even though the approach proposed in^6,8^ allows for modeling of the lithium intercalation into the active cathode material, the number of independent parameters of the model can be further decreased and thermodynamic consistency of the model can be further enhanced if anisotropy of the crystal lattice is introduced into the regular solution theory.

The second challenge that arises from the use of the regular solution theory for modeling Li intercalation into the active cathode material is related to the consideration of the active particle surface. Phase field modeling of Li intercalation dynamics unavoidably calls for four different boundary conditions^11^. At the phase boundary between electrolyte and active cathode material, the natural boundary condition is applied that interrelates the surface concentration (*c*) and the boundary surface free energy (*γ*)^23^. Two common approaches for implementing natural boundary conditions can be found in the literature. The first approach assumes constant surface energy, *γ*^6,7,10^, which is the simplest possible solution of the boundary problem. Nevertheless, recent experimental and theoretical studies^23^ show that such a description of the phase boundary between the cathode material and electrolyte is too oversimplified to efficiently describe all surface phenomena observed over the entire state of charge (SoC) range of the particle. Initiation of the phase transformation from Li-poor to Li-rich phases (nucleation) in phase separating materials mainly occurs at the particle surface, which cannot be adequately described by constant *γ*^23^. Cogswell *et al*.^23^ showed that with the use of non-constant surface energy, *γ*, in the model, the nucleation of phases on the surface can be described. This is in line with the second approach of natural boundary condition implementation that assumes linear dependency of *γ* on *c* over the entire SoC range of the particle. Non-constant *γ* leads to initiation of a spinodal decomposition reaction on the active particle surface, which corresponds to the experimental observation^24^. Nevertheless, the linear dependency of *γ* (a valid description of salt solution surfaces^25,26^) does not offer a fully thermodynamically consistent description of the interaction between the bulk and the surface of active cathode material^27^. This is because governing equations of the bulk dynamic are, unlike those for the surface dynamics, derived from the regular solution theory.

As regular solution theory plausibly describes lithium dynamics in the particle bulk^17–19^, elaboration of more adequate functional relations of *γ* represents an important contribution towards higher fidelity battery models. Nadkarni *et al*.^28^ (uploaded to arXiv; currently in recension) were the first to tackle this challenge in LFP material by the use of higher order polynomial dependency of *γ* vs. *c*. Functional dependency was obtained empirically by polynomial interpolation of the surface free energy values predicted by DFT calculations^29^. Therefore, the proposed methodology relies on a relatively complex workflow.

To provide a solution to the outlined challenges, this paper offers an innovative derivation of the chemical potential of Li in the active cathode nanoparticle from the regular solution theory. This derivation enables a mechanistic consideration of the anisotropy of the crystal lattice in parameters of the governing equation for the chemical potential. Moreover, application of the same theory enables derivation of thermodynamically consistent functional dependency of *γ* vs. *c*. Elaborated equations for the chemical potential and the surface free energy can be directly integrated into a particle model, which ensures a high level of predictiveness of the model. To validate plausibility of the newly developed approach, the derived theory was implemented for the LFP cathode material. The original contributions of the paper are therefore as follows:

- Explicit derivation of the chemical potential of Li in the active cathode nanoparticle and the interaction energies between diffusing species based on the regular solution theory by considering crystallographic parameters of the cathode material, which also enables mechanistic determination of the gradient penalty coefficient from the mixing enthalpy.
- Mechanistic derivation of phase boundary thickness between Li-rich and Li-poor phases in a phase separating cathode material.
- Thermodynamically consistent derivation of the surface free energy at the interface between the surface and the bulk of the particle from regular solution theory, where the surface of the active cathode particle is treated as a surface of the regular solution.
- A novel quasi-two-dimensional LFP particle modeling framework for modeling Li intercalation dynamics with the boundary condition and chemical potential derived from the regular solution theory.

## Methods

### Governing Equations

#### Cahn-Hilliard Equation

The temporal and spatial evolution of Li concentration inside active cathode particles is described by the Cahn-Hilliard equation. In general, the Cahn-Hilliard equation can be written as^1,10,30^:

1 $$\frac{\partial c({\bf{r}},t)}{\partial t}=\frac{1}{{k}_{b}T}\nabla D(c({\bf{r}},t))\nabla \mu (c({\bf{r}},t)),$$

where *k*_*b*_*T* is the thermal energy; $\nabla$ is the vector operator of spatial derivatives; *c* represents the non-dimensional concentration of lithium (usually non-dimensional molarity), which depends on two independent variables [time (t) and position (**r**)]; *D* is the diffusion coefficient, which is generally a function of *c*; and *μ* represents the chemical potential of lithium. The chemical potential *μ* inherently depends on *c*. In the limiting case of a constant diffusion coefficient and logarithmic dependency of the chemical potential on the concentration, the Cahn-Hilliard equation simplifies to Fick’s second law of diffusion. In the case of solid crystalline cathode material, the chemical potential is derived from the regular solution theory. The most general form of the chemical potential reads^2,30^:

2 $$\mu ={k}_{b}T\,\mathrm{ln}\,(\frac{c}{1-c})+{\rm{\Omega }}(1-2c)-\nabla (\kappa \nabla c)+B(c-\bar{c}),$$

where Ω is the enthalpy of mixing of diffusing species, *κ* is the gradient penalty between two phases in phase separation materials, and *B* is the phase boundary strain.

The regular solution enthalpy of mixing, Ω, and the gradient penalty parameter, *κ*, can be obtained from the regular solution theory (e.g.^1,2,17–20^), and the strain coefficient *B* can be obtained from the theory of elasticity (e.g.^10,30^). However, the present derivations of parameters Ω and *κ* did not take into account properties of the crystal lattice. In these approaches, crystallographic properties of the cathode material are introduced to the model empirically through the use of an anisotropic diffusion constant and the strain term *B*. This deficiency of present models is approached in the next section, where a thermodynamically consistent derivation of Ω and *κ* from the regular solution theory that considers crystallographic parameters of the cathode material is presented.

#### Enthalpy of Mixing and Gradient Penalty

The Cahn-Hilliard equation (Eq. 1) and the chemical potential (Eq. 2) are obtained from minimization of the total free energy functional, which can be derived from the regular solution theory (Appendix). The theory of non-uniform regular solution derived by Cahn and Hilliard^20^ can be applied to the crystalline cathode material by considering lithium atoms and vacancies in the crystal lattice as two diffusing species in the solid solution^19^. Regardless of the high anisotropy of cathode material crystal lattices, regular solution (designed for isotropic media in^16^) allows for description of lithium intercalation dynamics, which is also in agreement with the general consensus communicated in influential refs^17–19^, indicating that the regular solution description of anisotropic crystalline material is sufficiently relevant for elaborating state-of-the-art Li intercalation models.

Equations for the chemical potential and concentration dynamics of non-uniform regular solutions (Eqs 1 and 2) were first derived by Cahn and Hilliard^20^, and they led to the determination of parameters Ω and *κ*. Applied to crystalline cathode material, the original expressions of Ω and *κ* read^17–19^:

3 $${\rm{\Omega }}=\frac{Nz}{{N}_{A}{c}_{m}}(2{\varepsilon }_{Liv}-{\varepsilon }_{LiLi}-{\varepsilon }_{vv}),$$

4 $$\kappa =\frac{Nz{\lambda }^{2}}{2}(2{\varepsilon }_{Liv}-{\varepsilon }_{LiLi}-{\varepsilon }_{vv}),$$

where *c*_*m*_ denotes the maximal molarity of lithium in the cathode material, *N* represents the areal density of Li intercalation sites, *z* is the number of intercalation sites nearest to the reference site, and *N*_*A*_ is the Avogadro constant. Parameters *ε*_*LiLi*_, *ε*_*Liv*_ and *ε*_*vv*_ denote pair interaction energies of Li intercalation sites occupied by two lithium atoms, a lithium atom vacancy, and two lithium atom vacancies, respectively. In the theory of Cahn and Hilliard^20^ (Eqs 3 and 4), which was developed for isotropic solutions for application on liquid binary solutions, the quasi-crystal approximation was used^16^. The quasi-crystal approximation was first proposed by Guggenheim in the original ref.^16^ that postulated regular solution theory; since then, this approximation has been quickly adopted for describing amorphous media. An amorphous isotropic liquid regular solution is treated as an ordered closely packed crystal lattice with a well-defined number of nearest neighbors *z* and a crystal plane areal site density *N*. Two parameters of the crystal lattice are introduced to the chemical potential (*N* and *z*), although the theory is designed for non-crystalline media^20^. An additional parameter, *λ*, is needed for the mathematical consistency of the theory^20^. Parameter *λ* denotes the distance used for defining the concentration gradient in discretized media of the crystal lattice (usually a few inter-particle distances^20^). In the phase separating materials (i.e., LFP), *λ* can be interpreted as the thickness of the phase boundary between Li-rich and Li-poor phases^4,10,11,20,30^. From Eqs 3 and 4, the relation between Ω and *κ* is obtained:

5 $$\kappa =\frac{{\lambda }^{2}{c}_{m}{N}_{a}}{2}{\rm{\Omega }},$$

which is also used by the authors of refs^11,30^. In multiple refs^4,10^, parameter *κ* is determined by fitting parameter *λ* to the experimental observations.

As exposed in the Introduction, an innovative approach to deriving the chemical potential from regular solution theory includes explicit thermodynamically consistent determination of parameters Ω and *κ*, based on crystallographic properties and the interaction energies between diffusing species (lithium atoms and vacancies). This mechanistic evaluation of parameters Ω and *κ* from the partition function is presented in the Appendix: Eqs A.6 and A.7.

For parameter Ω, the same expression was obtained as that in ref.^20^ (Eq. 3), and parameter Ω thus explicitly depends on two crystallographic parameters: *N* and *z*. Unlike in other references (e.g.^4,10,11,30^), an innovative expression is obtained for parameter *κ*:

6 $$\kappa =\frac{Nz{d}^{2}}{2m}(2{\varepsilon }_{Liv}-{\varepsilon }_{LiLi}-{\varepsilon }_{vv}),$$

This expression includes parameter *d*, which represents the distance between crystal planes, and a newly introduced parameter *m*. Parameter *m* is explained in detail in the subsection Evaluation of Parameter *m*; similarly, to parameters *N* and *d*, it depends on the geometry of the crystal plane family under consideration.

The proposed approach therefore adequately reflects the crystal structure, including its potential anisotropy, in the parameters Ω and *κ*, and via Eq. 2, the crystallographic parameters are also adequately reflected in the chemical potential, which is an innovative contribution of this paper.

Resultingly, by combining Eqs 3, 5 and 6, parameter *λ* can be further determined as:

7 $$\lambda =\frac{d}{\sqrt{m}}.$$

Parameter *λ* depends on two parameters *d* and *m* (see subsection Evaluation of Parameter *m*), which are mechanistically derived from the crystal properties of the cathode material.

#### Boundary Conditions

The system of two equations, Eqs 1 and 2, represents a fourth-order partial differential equation system, which unavoidably calls for four boundary conditions:

8 $${F}_{bulk}={\frac{D}{{k}_{b}T}\nabla \mu |}_{bulk}=0,$$

9 $${\nabla c|}_{bulk}=0,$$

10 $${F}_{surface}={\frac{D}{{k}_{b}T}\nabla \mu |}_{surfce}={F}_{BV}$$

and

11 $${\kappa \nabla c|}_{surface}=\frac{\partial \gamma }{\partial c},$$

where *F* denotes the lithium flux, subscript *bulk* refers to the geometrical center of a particle, subscript *surface* refers to the particle surface, and *F*_*BV*_ denotes the Butler-Volmer flux, which is described by the Butler-Volmer equation^31,32^, the standard phenomenological description of electrode kinetics^33–36^. Two boundary conditions in the particle bulk (Eqs 8 and 9) are obtained from the symmetry of the problem. They are written for the geometrical center of the particle, where reflection symmetry must be satisfied in all dimensions. The first boundary condition for the particle surface (Eq. 10) consistently describes lithium flux through the particle surface from electrolyte. The second boundary condition on the particle surface (Eq. 11) includes surface effects related to the surface free energy of the particle surface in contact with the electrolyte. It is called the natural boundary condition. The challenge in determining this boundary condition (Eq. 11) arises from the unknown functional dependency of the surface free energy between particle and electrolyte on the lithium concentration. One of the innovative contributions of this paper thus arises from the explicit thermodynamically consistent derivation of the natural boundary condition in Eq. 11, which allows for its direct integration into the particle model.

#### Natural Boundary Condition

The natural boundary condition on the surface between LFP and electrolyte is obtained by minimization of the total free energy functional with application of the Lagrange variational principle^37^ (Appendix). The natural boundary condition implies proportionality between the derivative of the surface-energy concentration (∂*γ*/∂*c*) and the concentration gradient $\nabla c$ on the boundary (Eq. 12). The proportionality coefficient is equal to the magnitude of the gradient penalty term *κ*:

12 $${\kappa \nabla c|}_{\partial }=\frac{\partial \gamma }{\partial c},$$

where ∂ denotes the boundary of the calculation domain (surface of the particle).

Two simpler approaches with the constant *γ* value and the linear *γ*(*c*) dependency, which were addressed in the Introduction, result in the following boundary conditions^11^:

13 $${\kappa \nabla c|}_{\partial }=0\,{\rm{and}}\,{\kappa \nabla c|}_{\partial }=\beta ,$$

where *β* is a constant.

#### Constant $\tfrac{\partial \gamma }{\partial c}$ and Hildebrand formula

As addressed in the Introduction, the stability and accuracy of the models can be improved if the particle surface is considered as the surface of a regular solution. The surface of the regular solution was extensively studied in the case of liquid regular solutions by several authors^38–41^. Hildebrand showed from the Gibbs adsorption formula that the surface energy of the regular solution must satisfy the following condition^27^:

14 $$\frac{\partial \gamma }{\partial {c}^{\ast }}=\frac{{\rm{\Gamma }}}{{c}^{\ast }(1-{c}^{\ast })}[{k}_{b}T-{\rm{\Omega }}{c}^{\ast }(1-{c}^{\ast })],$$

where c^*^ denotes the equilibrium bulk concentration and Γ is adsorption, which measures how much lithium is adsorbed on the surface.

The Hildebrand relation (Eq. 14) is not satisfied for the most commonly used linear dependency of *γ*(*c*) (used in the boundary condition in Eq. 13), which reads:

15 $$\gamma (c)={\gamma }_{FeP{O}_{4}}(1-c)+{\gamma }_{LiFeP{O}_{4}}c,$$

where *γ* denotes the surface free energy of the mixture, ${\gamma }_{FeP{O}_{4}}$ and ${\gamma }_{LiFeP{O}_{4}}$ represent free surface energies of pure phases *FePO*_4_ and *LiFePO*_4_, and *c* denotes the lithium surface concentration. This leads to a conclusion that a more complex model of surface concentration dynamics should be incorporated in order to provide full thermodynamic consistency.

The special case of Eq. 15, where ${\gamma }_{FeP{O}_{4}}={\gamma }_{LiFeP{O}_{4}}$, results in constant *γ*(*c*), and consequently, ∂*γ*/∂*c* = 0. This *γ*(*c*) dependency satisfies Hildebrand’s condition for a regular solution in the case where no adsorption on the surface occurs, whereas it is subjected to the deficiencies addressed in the Introduction.

#### Surface free energy of the regular solution

The functional dependency of the surface free energy *γ*(*c*) of an active cathode particle surface in contact with electrolyte was derived by the Murakami approach^40^. Murakami *et al*.^40^ proposed derivation of the regular solution surface energy as a generalization of better known approaches of Guggenheim^38^ and Defay and Prigogine^39^. It is derived for the general case of an infinite surface of isotropic regular solution of the solvent with two mixing solutes and a vacuum. Murakami *et al*.^40^ used the quasi-crystal approximation for the liquid solution description, which approximates an amorphous liquid phase as an ordered quasi-crystal with well-defined positions of nearest neighboring atoms of the reference atom. Therefore, their theory can be generalized and applied also to the ordered crystalline solid solution.

An original contribution of this paper, addressed in the third bullet point of Introduction arises from the first successful application of the Murakami *et al*.^40^ approach, which was elaborated for the isotropic media that takes in consideration only nearest neighboring atoms, to the anisotropic crystal lattice. To consistently account for the phenomena in batteries, governing equations were derived for the crystal solid solution of lithium in LFP in contact with electrolyte. This resulted in explicit dependency of the surface free energy of the phase boundary between LFP and the electrolyte on the lithium surface concentration, given in the Appendix (Eq. A.5), since the areal integral part of the total free energy *F* is by definition equal to the surface free energy density *γ*. The functional dependency of the surface free energy between LFP and electrolyte thus reads:

16 $$\begin{array}{rcl}\gamma  & = & {\gamma }_{LiFeP{O}_{4}}\tfrac{c}{{c}_{m}}+{\gamma }_{FeP{O}_{4}}(1-\tfrac{c}{{c}_{m}})-m{\rm{\Omega }}\tfrac{c({c}_{m}-c)}{{c}_{m}^{2}}\\  &  & -\,\tfrac{1}{2{k}_{b}T}{[({\gamma }_{LiFeP{O}_{4}}-{\gamma }_{FeP{O}_{4}})+m{\rm{\Omega }}\tfrac{({c}_{m}-2c)}{{c}_{m}}]}^{2}\tfrac{c({c}_{m}-c)}{{c}_{m}^{2}},\end{array}$$

The first two terms in Eq. 16 represent the linear dependency, which is the same as in Eq. 15. In addition to the first two linear terms, Eq. 16 includes a quadratic Ω term, multiplied by parameter *m* (defined and explained in the next section). This term in Eq. 16 can be interpreted as a correction to the mixing enthalpy of the surface layer due to absent nearest neighbors. Higher-order terms in Eq. 16 arise from the changed ratio between the type of interacting species pairs (i.e., pair ratios of Li-Li, vacancy-Li and vacancy-vacancy) due to adsorption of lithium to the particle surface and the interaction of diffusing species with electrolyte.

**Figure 1:**
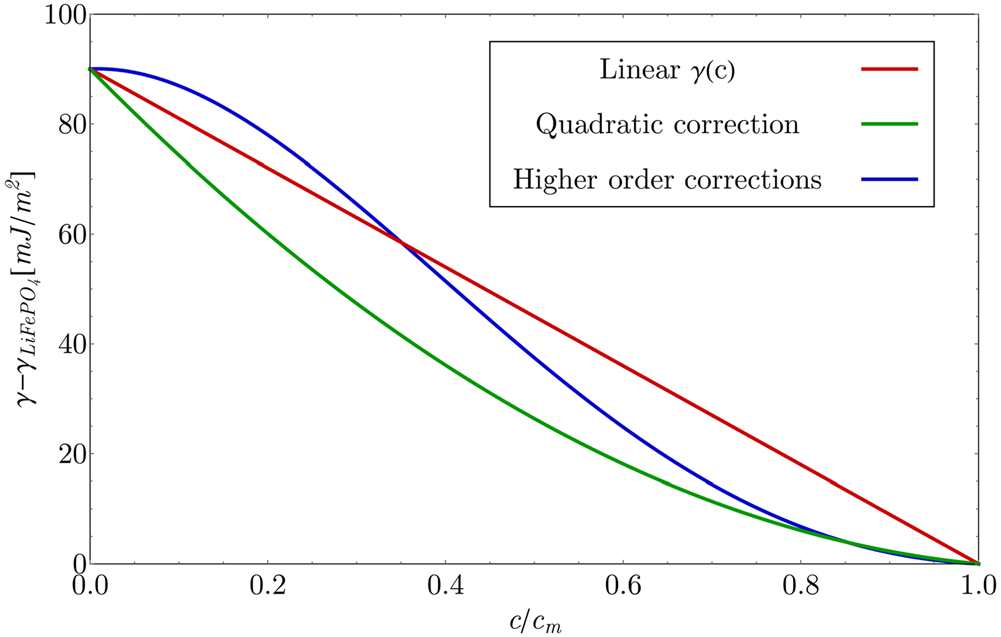
Functional dependency of LFP/electrolyte phase boundary surface free energy on the non-dimensional surface molarity of lithium relative to ${\gamma }_{LiFeP{O}_{4}}$. The dependency plotted in red represents the first two terms from Eq. 16. The first two terms and the quadratic correction are plotted in green, and the total *γ*(*c*) dependency from Eq. 16 with all higher order correction terms is plotted in blue.

Figure 1 represents the plot of surface free energy as a function of surface concentration, where separate contributions of different terms of Eq. 16 are presented. Parameters used in the Fig. 1 are the same as the ones used in model (Modeling section) and the difference ${\gamma }_{LiFeP{O}_{4}}-{\gamma }_{FeP{O}_{4}}=-\,90\,mJ/{m}^{2}$ is taken from the reference of Ferguson *et al*.^23^. Figure is plotted relative to the value of ${\gamma }_{LiFeP{O}_{4}}$.

The red line in Fig. 1 represent the first two terms of Eq. 16, which represent the linear dependency of *γ*(*c*) (Eq. 15). The green line in Fig. 1 shows the first two linear terms and the quadratic contribution of the third term. The blue line in Fig. 1 shows a full dependency of the surface free energy on the concentration in Eq. 16. The differences between the linear *γ*(*c*) function (red line in Fig. 1) and the newly derived *γ*(*c*) dependency (blue line in Fig. 1) is most pronounced at high and low surface concentration (beneath the first spinodal point and above the second spinodal point). Therefore, the new thermodynamically consistent derived *γ*(*c*) dependency most severely enhances the lithium dynamics near the surface in the solid solution regime, since the slope of the function *γ*(*c*) is most severely altered by the correction terms in these two regions. This enables consistent description of Li surface dynamics over the entire SoC range, since newly derived explicit *γ*(*c*) dependency does not initiate the phase transition in LFP prior to reaching the spinodal concentration, which is in agreement with experimental observations^23,24^.

#### Evaluation of Parameter *m*

Equations 5, 6 and 16 include the explicit dependency on a newly introduced parameter *m* (derivation in Appendix). Model parameter *m* was determined from the crystallographic properties of *LiFePO*_4_.

In the classical quasi-crystal formulation of regular solution theory, only interactions between nearest neighbors are taken into account^38–40^. When applying regular solution theory to the description of the solution surface, absent nearest neighbors on the surface are the main contribution to the surface energy^38^. The regular solution model (Eqs A.1 and A.3 in Appendix) accounts for this contribution through the mechanistically based parameter *m*. *m* represents the half fraction of the nearest neighbors outside the crystallographic plane parallel to the surface, and its product with the number of nearest neighbors coincides with the number of absent nearest neighbors at the surface. *m* is thus a measure of the deficit in interaction energy due to the absent nearest neighbors at the surface.

**Figure 2:**
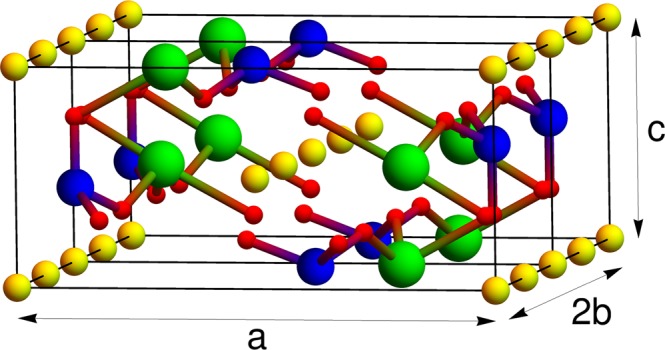
Two unit cells of *LiFePO*_4_ crystal lattice. Green spheres represent Fe atoms; blue spheres, P atoms; yellow spheres, Li atoms; and red spheres, O atoms.

Due to the high anisotropy of the *LiFePO*_4_ crystal lattice (Fig. 2), every Li site in the crystal only has two nearest neighbors (in *b* direction, Fig. 2). The contributions of all other Li crystal sites to the interaction energy are thus not taken into account if classical quasi-crystal formulation of regular solution theory is applied to the anisotropic crystal lattice.

**Figure 3:**
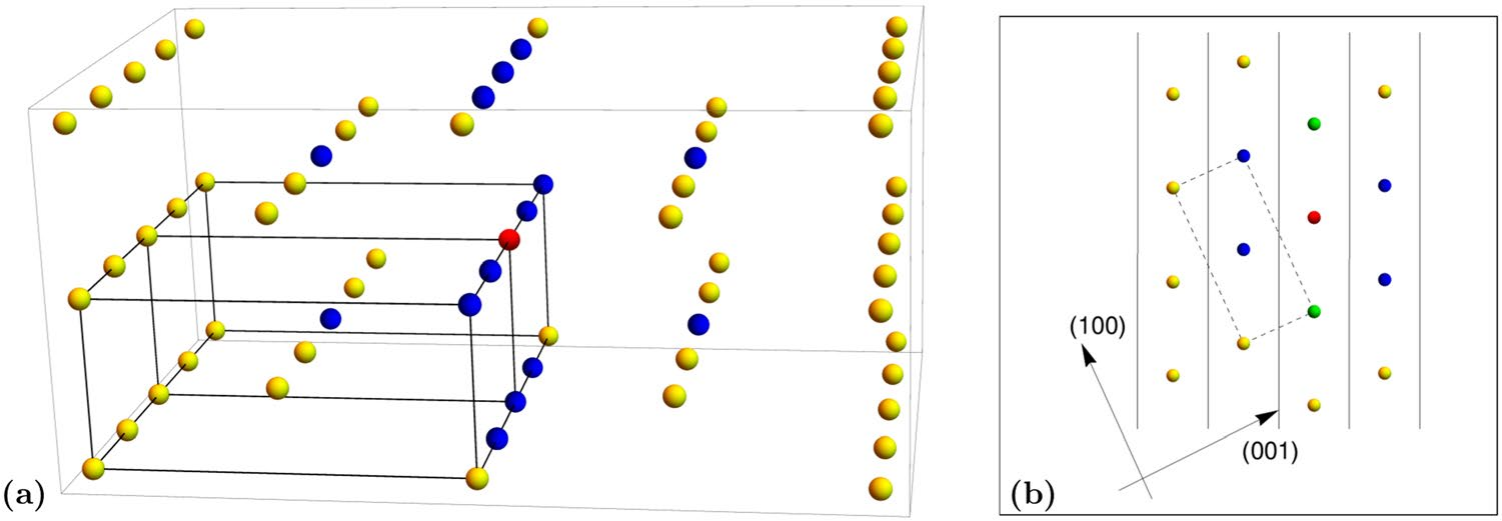
(**a**) Isolated Li-intercalation sites (represented by yellow, blue, and red spheres) in *LiFePO*_4_ crystal. The red sphere represents the reference Li-site, and the blue spheres represent the fourteen nearest neighbors (neighbors in the radius of 0.61 *nm*). The black prisms show two unit cells of the *LiFePO*_4_ crystal, the same as in Fig. 2. (**b**) Shows a projection of Li-intercalation sites in a Bravais lattice on the (010) plane. Vertical lines represent (101) crystal planes in the perpendicular projection. In-plane neighbors to the red-marked reference Li-site are marked with green, while the out-of-plane neighbors are marked with blue. Yellow Li-sites are Li-sites outside a 0.61 *nm* radius. The dashed rectangle shows a projection of a *LiFePO*_4_ crystal lattice unit cell.

To solve this challenge, a modification of the existing approach is proposed in this paper. The meaning of parameter *m* from the quasi-crystal formulation of regular solution was generalized in order to consider contributions of all Li crystal sites to the total interaction energy. To preserve the main significance of parameter *m* (i.e., a measure of the deficit in interaction energy due to the absent nearest neighbor at the surface), an innovative generalization of *m* for the anisotropic lattice was designed. Parameter *m* was determined as a sum of normalized individual contributions of all neighbors that lie outside of the lattice plane parallel to the surface (lattice sites marked blue in Fig. 3(b)).

Li intercalation sites (yellow spheres in Fig. 3(a)) themselves form a hexagonal crystal lattice. Pair interactions that depend only on the distance between two particles can be calculated for sites of such lattices. The magnitude of the contribution of an individual Li site to the interaction energy decreases with distance. The total interaction energy is obtained by spatial integration of the pair interaction potential (*ϕ*(*r*)) multiplied by the pair distribution function (*w*(*r*))^42^

17 $$ {\mathcal F} ={\int }_{0}^{\infty }\,\varphi (r)w(r)dr.$$

For the pair interaction *ϕ*(*r*), the Lennard-Jones potential was used: *ϕ*(*r*) = *ε*[(*r*_*m*_/*r*)^12^ − (*r*_*m*_/*r*)^6^], where constants *ε* and *r*_*m*_ determine strength of the potential (depth of minimum) and position of nearest neighbor (position of the minimum). Parameter *r*_*m*_ is known and accounts for the *r*_*m*_ = 3.004 *Å* (2). The pair distribution function in the crystal coincides with the electron density, which can efficiently be described by the summation of Dirac delta functions at Bravais lattice points (Li-intercalation sites): $w(r)={\sum }_{j}\,\delta (r-{R}_{j})$, where *R*_*j*_ represent the positions of Li-intercalation sites in the *LiFePO*_4_ crystal lattice. The integral in Eq. 17 therefore, transforms to the summation over the contributions of individual Li-intercalation sites to the total interaction energy:

18 $$ {\mathcal F} =\sum _{j=1}^{14}\,{ {\mathcal F} }_{j}=\sum _{j=1}^{14}\,\varepsilon [{(\frac{{r}_{m}}{{r}_{j}})}^{12}-{(\frac{{r}_{m}}{{r}_{j}})}^{6}].$$

The calculation of normalized contributions of each Li-intercalation site to the total interaction energy shows that a non-negligible contribution to the pair interaction comes from pairs that are less than 0.61 *nm* apart. The contribution of the pairs that are 0.61 *nm* apart is on the order of a percent, whereas the contribution of the next furthest pairs is an order of magnitude smaller. Every Li site has fourteen neighbors within a 0.61 *nm* radius (Fig. 3(a)), which is the upper summation limit in Eq. 18. The normalized contributions of all fourteen nearest neighbors to the interaction energy are listed in Table 1.

**Table 1:** Normalized contribution of an individual lattice neighbor to the total pair interaction energy.

Neighbor direction	distance *Rj*	Multiplicity	${{\boldsymbol{ {\mathcal F} }}}_{{\boldsymbol{j}}}$/${\boldsymbol{ {\mathcal F} }}$
(0, 1, 0)	3.004 Å	2	0.371
(0, 0, 1)	4.692 Å	2	0.049
(0, 1/2, 1/2)	5.571 Å	4	0.018
(1/2, 0, 1/2)	5.675 Å	4	0.016
(0, 2, 0)	6.008 Å	2	0.011

*m* was calculated for the surface parallel to the crystal planes with the orientation of (101). This is because (101) is the crystal orientation that shows the lowest misfit between *LiFePO*_4_ and *FePO*_4_ crystals^10^ (lowest parameter *B* in Eq. 20). As a consequence, all phase boundaries between *LiFePO*_4_ and *FePO*_4_ are oriented normal to the 101 direction due to strain energy minimization^10^. From the projections in Fig. 3(b), the type and number of Li-site neighbors inside and outside the crystal plane parallel to the surface can be determined, which defines parameter *m* (blue and green spheres in Fig. 3(b)) as:

19 $$m=\sum _{j=1}^{5}\,\frac{{ {\mathcal M} }_{j}{ {\mathcal F} }_{j}}{ {\mathcal F} }$$

Parameter ${ {\mathcal M} }_{j}$ in Eq. 19 is the number of atoms that lay outside the crystal plane for each of five crystallographic direction of neighbors inside 0.61 nm distance (determined from the Fig. 3). For the calculated (101) orientation of the LFP that is simulated in proposed single particle model (Eqs 20, 21 and 23), *m* = 0.101. The elaborated methodology is applicable also to other crystallographic orientations of the surface. Table 2 shows values of parameters *m* for five differently oriented surfaces.

**Table 2:** Parameter *m* for different crystallographic orientations of crystal surface.

Lattice plane	*m*
{0, 1, 0}	0.382
{1, 0, 0}	0.034
{0, 1, 1}	1
{1, 0, 1}	0.101
{2, 0, 1}	0.118

## Modeling

**Figure 4:**
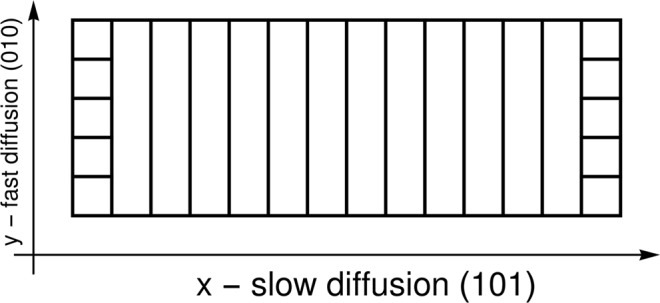
Discretization of the calculation domain.

To validate the plausibility of the newly developed approach for determining the chemical potential based on the regular solution theory by considering the crystallographic parameters of the LFP cathode material (Eqs 2, 3 and 6) and the functional dependency of surface free energy on lithium concentration (Eq. 16), a simulation model was developed. Phase field modeling of lithium intercalation was performed in one dimension ((101) crystal direction in LFP) with additional discretization of surface planes in the perpendicular direction ((010) crystallographic direction) for accurate implementation of newly derived surface phenomena (the discretization of the computational domain is schematically shown in Fig. 4). This forms a basis of a quasi-two-dimensional model that represents a good trade-off between model complexity, computational demand and relevance of the results of the model. Nevertheless, the derived methodology is universal, and it can be used for modeling Li intercalation into LFP within a modeling framework of arbitrary dimensionality.

The Cahn-Hilliard equation was solved in the two-dimensional domain for the Li intercalation simulation:

20 $$\tfrac{\partial c}{\partial t}=\tfrac{{D}_{0}}{{k}_{b}T}\nabla \tfrac{({c}_{m}-c)c}{c}\nabla \mu +\tfrac{1}{n{e}_{0}}{I}_{0}({c}_{m}-c){e}^{\alpha \mu /{k}_{b}T}({e}^{-{e}_{0}\alpha \eta /{k}_{b}T}-{e}^{(1-{e}_{0}\alpha \eta /{k}_{b}T)})$$

with

21 $$\mu ={k}_{b}T\,\mathrm{ln}(\frac{c}{{c}_{m}-c})+{\rm{\Omega }}(\frac{1-2c}{{c}_{m}})-\frac{\kappa {V}_{s}}{{c}_{m}}{\nabla }^{2}c+\frac{B}{{N}_{A}{c}_{m}}(\frac{c-\bar{c}}{{c}_{m}}).$$

As elaborated in the Governing Equations section, four boundary conditions were needed for the solution of a fourth-order PDE system. Concentration and chemical potential gradients were set to zero at the center of the particle due to the symmetry of the problem. At the surface parallel to the (101) crystal planes, Butler-Volmer flux in the *x* direction was defined the gradient of chemical potential. The concentration gradient on the particle surface was determined from calculated *γ*(*c*) dependency (Eq. 16). The surface flux across the (010) oriented surface (direction of fast diffusion) was modeled by the source term (Allen-Cahn model^3–5^) in Eq. 20. For this specific case, the boundary conditions in (Eqs 8–11) are:

22 $${\nabla c|}_{x=0}=0,\,{\nabla \mu |}_{x=0}=0,$$

23 $${\hat{{\bf{n}}}\kappa \nabla c|}_{x=L/2}={c}_{m}^{2}\frac{\partial \gamma }{\partial c},\,{\nabla \mu |}_{x=L/2}=\frac{{k}_{b}T{c}_{m}}{{D}_{xx}n{e}_{0}}{I}_{0}{e}^{\alpha \mu /{k}_{b}T}({e}^{-{e}_{0}\alpha \eta /{k}_{b}T}-{e}^{(1-{e}_{0}\alpha \eta /{k}_{b}T)}).$$

All model parameters and constants of the model are listed in Table 3.

**Table 3:** Quantities used in the model.

Symbol	Quantity	Value	References
*c*	molarity of Li	dependent variable	
$\bar{c}$	average molarity of Li	${\int }_{V}\,cdV/{\int }_{V}\,dV$	
*μ*	chemical potential	dependent variable	
*t*	time	independent variable	
*D*_0_	Two dimensional diffusion coefficient tensor	$$[\begin{array}{cc}{D}_{xx} & 0\\ 0 & {D}_{yy}\end{array}]$$	
*D*_*xx*_	diffusion coefficient in (101) direction	8 × 10^−16^ *m*^2^/*s*	^30^
*D*_*yy*_	diffusion coefficient in (010) direction	4 × 10^−13^ *m*^2^/*s*	^46^
*c*_*m*_	maximal molarity of Li	22900 *mol*/*m*^3^	^11^
Ω	regular solution parameter	0.115 *eV*	^11^
*κ*	gradient penalty coefficient	2.453 × 10^9^ *eV*/*m*	^11^
*V*_*s*_	intercalation site volume	0.218 *nm*^3^	^4, 12^
*V*	particle volume	2.5 × 10^5^ *nm*^3^	
*B*	strain in (101) direction	0.19 *GPa*	^30^
*α*	charge transfer coefficien	0.5	^47^
*η*	overpotential	1 *mV*, 2 *mV* and 20 *mV*	
*ϕ*_*s*_ − *ϕ*_*l*_	potential difference across the particle-electrolyte interface	${V}_{OC}-\tfrac{\mu }{{e}_{0}}$	^48, 49^
*V*_*OC*_	open circuit voltage	3.422 *V*	^50^
*I*_0_	Butler-Volmer exchange current	1.6 × 10^−18^ *A*	^51^
*T*	temperature	300 *K*	
*L*	particle length in (101) direction	100 *nm* and 250 *nm*	
$\hat{{\bf{n}}}$	unit vector normal to the particle surface	$\tfrac{1}{\sqrt{{a}^{2}+{c}^{2}}}(a,0,c)$	
*n*	*Li*^+^ ion charge number	1	
*N*_*A*_	Avogadro constant	6.022 *mol*^−1^	
*k*_*b*_	Boltzmann constant	8.617 × 10^−5^ *eV*/*K*	
*e*_0_	elementary charge	1.602 × 10^−19^ *As*	

## Results and Discussion

**Figure 5:**
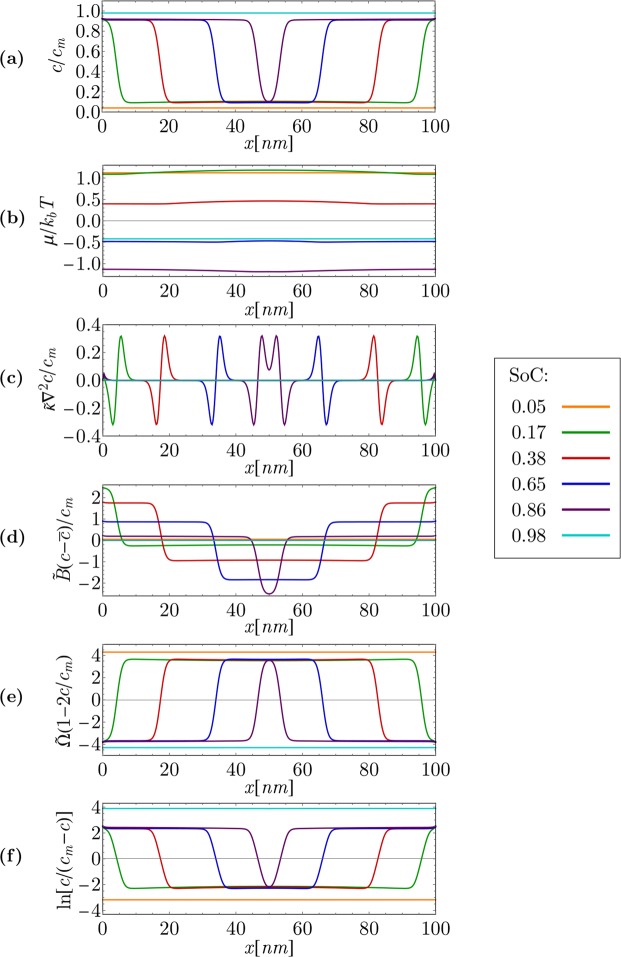
Low current Li intercalation in particle with 100 *nm* length at overpotential *η* = 1 *mV*. (**a**) Non-dimensional molarity of lithium spatial dependency, (**b**) chemical potential spatial dependency, (**c**) gradient penalty contribution to chemical potential, (**d**) strain energy contribution to chemical potential, (**e**) enthalpy of mixing contribution to chemical potential, and (**f**) entropy contribution to chemical potential. Rescaled parameters used in the axis labels can be written as follows: $\tilde{\kappa }=\tfrac{\kappa {V}_{s}}{{c}_{m}}$, $\tilde{{\rm{\Omega }}}=\tfrac{{\rm{\Omega }}}{{k}_{b}T}$, and $\tilde{B}=\tfrac{B}{{N}_{A}{c}_{m}}$.

**Figure 6:**
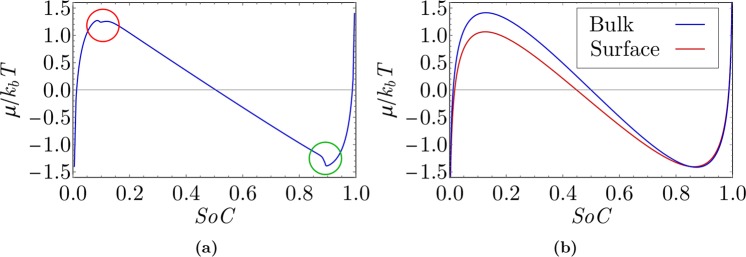
(**a**) Chemical potential dependency on SoC (spinodal curve) obtained by monitoring average chemical potential in the simulation of near equilibrium Li intercalation (*L* = 100 *nm*, *η* = 1 *mV*). (**b**) Chemical potential of the bulk (blue line) and surface (red line) during thermodynamically equilibrium charging.

**Figure 7:**
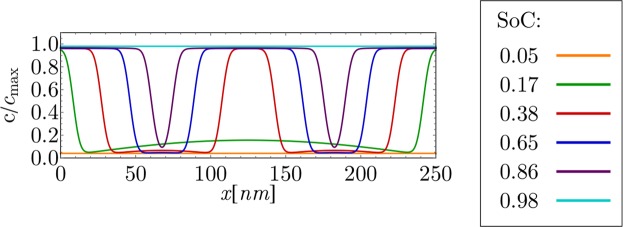
Near equilibrium, low current Li intercalation in particle with 250 *nm* length at overpotential *η* = 2 *mV*.

**Figure 8:**
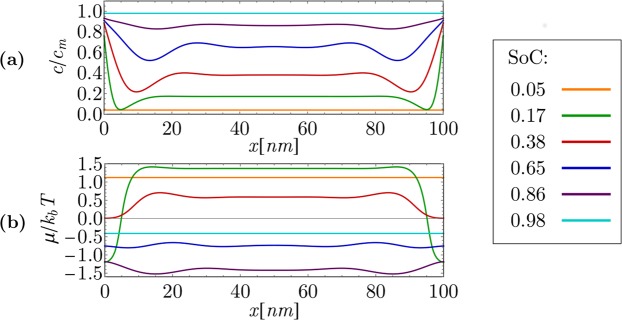
Non-equilibrium, high-current Li intercalation in particle with 100 *nm* length at overpotential *η* = 20 *mV*. (**a**) Lithium concentration, (**b**) chemical potential.

The results are presented as spatial plots of the non-dimensional lithium molarity (*c*/*c*_*m*_) and the one-dimensional chemical potential. The *x*-axis represents the (101) crystallographic direction. All simulations were performed for constant overpotential *η*. Results are presented on two different particle sizes (100 *nm* and 250 *nm* particles), since this are the representative particle sizes in commercial LFP cathodes^43,44^. Many different physicochemical phenomena can be seen from the results presented in Figs 5, 6, 7 and 8.

Figure 5 shows a simulation of near thermodynamic equilibrium of Li intercalation. Figure 5(a) shows the non-dimensional Li molarity, and Fig. 5(b) displays the corresponding chemical potential. Figure 5(c–f) show separate terms from the chemical potential (Eq. 21) at different SoCs of the particle. This corresponds to temporal evolution of concentration and chemical potential inside a 100 *nm* particle for the case of a near-equilibrium lithium intercalation from electrolyte to particle at a low overpotential of *η* = 1 *mV*.

Lithium intercalation to the active particle undergoes a solid solution regime until the first spinodal point is reached (Fig. 6). When a spinodal point is reached, initiation of the phase transition from *FePO*_4_ to *LiFePO*_4_ occurs at the particle surface, which is in agreement with findings in other references (e.g., Cogswell *et al*.^23^). The phase separation nature of the LFP material is well represented in Fig. 5(a). After the nucleation of the Li-rich phase at the particle surface, the phase field is decomposed into two regions: regions with high lithium molarity (*LiFePO*_4_ phase) and low lithium molarity (*FePO*_4_ phase).

The simulation in Fig. 5 was conducted for a very low overpotential, which produces near thermodynamic equilibrium conditions. Very low overpotential *η* yields a very low flux of lithium from the electrolyte to the particle bulk. Both phenomena are clearly reflected also in a very small spatial variation in the chemical potential inside the particle at a particular SoC value (Fig. 5(b)). Consequently, the temporal evolution of the Li concentration field in a particle follows a trend characteristic for near thermodynamic equilibrium conditions of the LFP material, which for a 100 *nm* particle (Fig. 5) results in domino cascade dynamics^4,9^, where two phase boundaries travel from the particle surface to the inside of the particle i.e., perpendicular to the fast diffusion direction ((010) crystallographic direction).

To clearly demonstrate spinodal decomposition in a particle, the particle-averaged value of the chemical potential obtained by integration over the entire computational domain is plotted as a function of a SoC of the particle (Fig. 6). The first fact that can be derived from the spinodal curve in Fig. 6(a) is that the phase transformation initiates at spinodal composition, which can be seen as a shallow local minimum at the top of the left spinodal potential barrier (the part of the plot in Fig. 6(a) at *SoC* = 0.128 marked with red circle). At the moment of *LiFePO*_4_ phase nucleation on the surface, the transient phenomenon of quick lithium adsorption to the surface occurs. A phase boundary emerges inside the particle bulk at this moment, which results in an additional contribution to the chemical potential due to strain and gradient penalty (Eq. 2 and 5(c) and (d)). In a very small SoC window (approximately from SoC of 0.128 to 0.132), a few crystal planes at the (101) surface are far from thermodynamic equilibrium (Fig. 6(a), although the entire particle is in near thermodynamic equilibrium conditions.

This observation can be explained also on the basis of a comparison between chemical potential of the bulk and the surface, calculated only from contributions of the entropy and the mixing enthalpy term of Eq. 2. Figure 6(b) shows chemical potentials of the bulk (blue plot in Fig. 6(b)) and the surface (red plot in Fig. 6(b)). Both were calculated from the entropy and the mixing enthalpy, since these two terms are the only ones that are explicitly dependent on *c*. An additional term that accounts for surface effects was introduced as the surface chemical potential (red plot in Fig. 6(b)) that was derived from Eq. 16. The maximum value of the chemical potential at the surface is reached prior to the bulk (lower value of red curve in comparison to blue curve at *SoC* = 0.128 in Fig. 6(b)). This explains adsorption of lithium to the particle surface.

At the second spinodal point, collision of intercalation waves^4^ can be seen in Fig. 6(a) (marked with a green circle). It can be seen in Fig. 5(a) that at this moment, two intercalation fronts meet at the center of the particle. The gradient penalty and strain terms drop to zero, which leads to a rapid decrease in the chemical potential, which is discernible in Fig. 6.

To investigate the significance of contributions of the gradient penalty, strain, enthalpy of mixing and entropy to the chemical potential (Eq. 21), separate contributions of each term in Eq. 21 are plotted in Fig. 5(c–f). The chemical potential consists of four different terms (Eq. 21): (1) the entropy term (${k}_{b}T\,\mathrm{ln}\,[c/({c}_{m}-c)]$, Fig. 5(f)) itself governs the diffusion of species in the system^45^; (2) the enthalpy of mixing (${\rm{\Omega }}\,[1-2c/{c}_{m}]$, Fig. 5(e)) accounts for the uphill diffusion effect, which is a prerequisite for modeling phase separation^1^; (3) the strain term ($B\,[(c-\bar{c})/{c}_{m}]/({N}_{A}{c}_{m})$, Fig. 5(d)) ensures that the content of lithium in the Li-poor phase as well as the content of vacancies in the Li-rich phase is at the solid solubility limit^10^; and (4) the gradient penalty term ($\kappa {V}_{s}/{c}_{m}{\nabla }^{2}c$, Fig. 5(c)) determines the thickness of the phase boundaries^4,10^.

Figure 5(c–f) very clearly show positions of phase boundaries between both the present phases as well as a non-zero thickness of such phase boundaries. The thickness of phase boundaries between Li-rich and Li-poor phases was estimated in two different ways. It was calculated for the (101) crystallographic orientation of phase boundaries from Eq. 7. The calculated value was compared to the one obtained from a simulation in which the phase boundary thickness is recognized as the distance between an adjacent minimum and maximum in a gradient penalty contribution to the chemical potential (Fig. 5(c)). A plausible value of *λ* = 2.7 *nm* was obtained by both approaches, which confirms adequacy of Eq. 7. The phase boundary thickness thus accounts for approximately five crystal planes. Parameter *m* can, therefore, also be interpreted as an inverse square of the number of crystal planes in the phase boundary between Li-rich and Li-poor phases (Eq. 7).

Figure 7 shows the results for a near equilibrium lithium intercalation at *η* = 2 *mV* for a larger 250 *nm* particle. Similar physicochemical phenomena, as already discernible in Fig. 5, can also be seen in Fig. 7. A phase transition is initiated at the particle surface. After that, Li-rich phase regions advance from the surface to the particle bulk in the domino cascade manner. Due to the larger particle size, in comparison to the particle from Fig. 5, another Li-rich phase region emerges at the center of the particle. In this way, four phase boundaries are created inside the particle. The newly emerged Li-rich region has the shape of a bend parallel to the (101) crystal plane, which is in agreement with the model of Cogswell *et al*.^10^. The thickness of Li-rich bands increases with time in the domino cascade manner due to the low overpotential.

At a high overpotential of *η* = 20 *mV* and a particle size of 100 *nm* (Fig. 8), the magnitude of the Butler-Volmer term in Eq. 20 exceeds the magnitude of the first diffusion term, and phase separation in the particle during lithium intercalation is suppressed. Li flux from electrolyte to the particle bulk through the (010) crystallographically oriented particle surface is large in comparison to the transverse uphill diffusion (Cahn-Hilliard dynamics in x direction in Fig. 8). Consequently, the phase field of Li concentration remains nearly constant. This coincides well with the results of *in*-*situ* X-ray diffraction measurements performed and published by Lim *et al*.^24^. On the other hand, the spatial dependency of the chemical potential is far from a nearly constant value in this case, indicating the non-equilibrium nature of lithium intercalation at this overpotential. The transition from the solid solution regime to the phase separation regime therefore does not occur in this case.

The presented results thus confirm that the proposed novel thermodynamically consistent derivation of the chemical potential of Li in an active cathode nanoparticle (which includes innovatively derived terms of gradient penalty, phase boundary thickness between Li-rich and Li-poor phases, and surface free energy at the interface between surface and bulk of the particle) allows for adequate simulation representation of recent experimental findings in the LFP material. In addition, the mechanistic basis of governing equations enables the achievement of credible simulation results over the entire SoC range, for arbitrary charging and discharging rates and particle sizes, confirming their general applicability. Furthermore, explicit analytic derivation of all equations allows for their direct integration into the particle model, which is another advantage of the proposed approach.

This is also one of the main strengths of the newly developed approach that motivates future applications, as electrodes consisting of phase separating materials are subjected to inter- and intra-particle phase separation being governed by a complex interplay between lithiation levels of particles, particle sizes and their potentials, and thus direct integration of the proposed governing equations in the multi-particle models allows for obtaining new insights also on the electrode level. This is of particular importance for more profound analyses of transport phenomena on the electrode and on the particle level, which are directly related to degradation phenomena driven by mechanical stresses due to inter- and intra-particle phase separation. Such type of applications thus pave the way towards simulation supported engineering of electrodes with enhanced performance and prolonged service life.

## Supplementary information

Appendix
