# Supplementary material for: Thermodynamically consistent derivation of chemical potential of a battery solid particle from the regular solution theory applied to LiFePO4
Source: Sci Rep. 2019 Feb 14;9:2123. doi: 10.1038/s41598-019-38635-2 (PMC6375981; doi:10.1038/s41598-019-38635-2)
Supplement: Supplementary file 1 — Appendix [file 41598_2019_38635_MOESM1_ESM.pdf]

Thermodynamically consistent derivation of chemical potential of a battery solid particle from the regular solution theory applied to $LiFePO_4$

Klemen Zelič[1,*] and Tomaž Katrašnik[1]

[1]University of Ljubljana, Faculty of Mechanical Engineering, Laboratory for Internal Combustion Engines and Electromobility, Ljubljana, SI-1000, Slovenia
[*]klemen.zelic@fs.lj-uni.si

Appendix

The Cahn-Hilliard equation (Equation 20), the chemical potential (Equation 21) and the natural boundary condition (Equation 11) describing Li intercalation in a crystalline cathode particle were obtained by minimization of the total free energy functional. The total free energy was obtained by constructing a partition function based on the regular solution theory[1]. The partition function constructed by the Murakami approach[1] includes all interaction energy contributions between diffusing species from the bulk and the surface of a regular solution. With the introduction of parameter m (explained in subsection Evaluation of Parameter m), crystallographic properties are brought to the partition function. The total free energy of the system derived from such a partition function therefore adequately describes both the bulk and the surface of the particle. The partition function of the system can be written as the sum of the energies of all possible states of the system:

$$Z = \overbrace{\left[\exp\left(\frac{z\varepsilon_{vv}+2\mu_v}{2kT}\right)\right]^{N_v+N_{Li}}}^{(A)} \sum_{y_i x_i} \overbrace{\exp\left[\frac{Nazmx_1(\varepsilon_{LiLi}+y_1\varepsilon_{vv}+Na(x_i\varepsilon_{LiE}-y_i\varepsilon_{vE}))}{2kT}\right]}^{(B)} \times$$
$$\times \prod_{i=1}^{\Lambda} \underbrace{\frac{(Na(x_i+y_i))!}{(Nax_i)!(Nay_i)!}}_{(C)} \underbrace{\exp\left[\frac{2Nax_i(\mu_{Li}-\mu_v)+Na(\varepsilon_{LiLi}-\varepsilon_{vv})}{2kT}\right]}_{(D)} \underbrace{\exp\left[-\frac{Nazp^i_{Liv}\Delta\varepsilon}{4kT}\right]}_{(E)}, \quad (A.1)$$

where T is temperature; k_b is the Boltzmann constant; subscripts v and Li denote vacancies and lithium, respectively; and index i counts the crystal planes parallel to the surface ((101) crystal planes). The counting of crystal planes starts at the surface. The highest possible index i is Λ, which denotes the total number of (101) crystal planes in the system (the length of the particle divided by the distance between planes). x_i and y_i denote the non-dimensional molarities of lithium and vacancies in the i-th crystal plane parallel to the surface ($x_i = c/c_m$, $y_i = 1 - c/c_m$). Since regular solution theory is applied to the crystal lattice with the fixed number of potential intercalation sites, the following condition exists:

$$x_i = 1 - y_i. \quad (A.2)$$

Further, ε_{LiE} and ε_{vE} represent interaction energies of a lithium atom and a lithium vacancy, respectively, with electrolyte. N represents the number density of lithium intercalation sites inside the crystal planes parallel to the surface. a is the area of the crystal planes parallel to the surface. N_v and N_{Li} are the number of vacancies and lithium atoms in the system. The sum of these two parameters gives the total number of Li intercalation sites in the particle. μ_v and μ_{Li} are chemical potentials of the vacancies and lithium, respectively. The chemical potential used in the model is defined as $\mu = \mu_{Li} - \mu_v$. $\Delta\varepsilon$ represents the interaction energy due to mixing of diffusing species, and it is defined as: $\Delta\varepsilon = 2\varepsilon_{Liv} - \varepsilon_v - \varepsilon_{Li}$. z is number of nearest neighbors in the crystal lattice. p^i_{Liv} represents the regular solution interaction with the nearest neighbors (Figure 3). p^i_{Liv} can be written as:

$$p^i_{Liv} = \begin{cases} x_i[my_{i-1}+(1-2m)y_i+my_{i+1}]+y_i[mx_{i-1}+(1-2m)x_i+mx_{i+1}] & i>1 \\ x_1[(1-2m)y_1+my_2]+y_1[(1-2m)x_1+mx_2] & i=1 \end{cases} \quad (A.3)$$

Equation A.1 can thus be interpreted as the following. The first term in the partition function Z (Term (A) in Equation A.1) describes the energy of a de-lithiated particle (only vacancies are present). The parts of the function Z under the summation

describe the free energy contribution of lithium and Li-vacancy mixing. The first term under the sum (Term (B) in Equation A.1) represents the deficit of interactions due to the surface and Li-sites interaction with electrolyte. The second exponential term (Term (D) in Equation A.1) includes Li-Li interaction. The last term (Term (E) in Equation A.1) represents the free energy of mixing. All possible constellations are taken into account by multiplication over all crystal planes (i from 1 to Λ) and summation over all possible concentration profiles at the fixed concentrations x and y.

The total free energy of the system was obtained from the partition function:

$$F = k_b T \ln Z. \tag{A.4}$$

The maximum term method[2] was used to eliminate summations over x_i ad y_i and to evaluate the partition function. The logarithm of the factorial term was approximated by the Stirling formula ($\ln n! \approx n \ln n - n$ for large n). Since Λ is large (the length of the crystal is large in comparison to the distance between crystal planes), the summation over i can be transformed in the integral, and continuous (phase field) representation is obtained. Inserting Equation A.1 in Equation A.4 and considering equations A.3 and A.2 yields:

$$F = \int_V \left[k_b T \frac{c}{c_m} \ln\left(\frac{c}{c_m}\right) + k_b T \left(1 - \frac{c}{c_m}\right) \ln\left(1 - \frac{c}{c_m}\right) + \frac{Nz\Delta\varepsilon}{N_A c_m} \frac{\overbrace{c(c_m - c)}^{(A)}}{c_m^2} + \frac{Nzd^2\Delta\varepsilon}{2c_m m} \overbrace{(\nabla c)^2}^{(B)} \right] dV +$$
$$+ \int_A \underbrace{\left\{ \gamma_{LiFePO_4} \frac{c}{c_m} + \gamma_{FePO_4}\left(1 - \frac{c}{c_m}\right) - m\frac{Nz\Delta\varepsilon}{N_A c_m} \frac{c(c_m - c)}{c_m^2} - \frac{1}{2k_b T}\left[(\gamma_{LiFePO_4} - \gamma_{FePO_4}) + m\frac{Nz\Delta\varepsilon}{N_A c_m}\frac{(c_m - 2c)}{c_m}\right]^2 \frac{c(c_m - c)}{c_m^2} \right\}}_{\gamma(c)} dA, \tag{A.5}$$

where d represents the distance between crystal planes parallel to the surface.

Equation A.5 consists of two separate contributions: bulk and surface. The first term (volume integral) represents the bulk, and the second term (surface integral) represents the surface. The volume integral represents the total free energy of the particle bulk. The first and second terms in under the volume integral represent entropy contribution to the total free energy. The third and fourth terms under the volume integral in Equation A.5 are obtained from p^i_{Liv}. The bulk part of p^i_{Liv} (Equation A.3 for $i > 1$) can simply be decomposed to terms $x_i y_i$ (recognized as Term (A) in Equation A.5) and $y_i(x_{i-1} - 2x_i + x_{i-1}) + x_i(y_{i-1} - 2y_i + y_{i-1})$. After transition to continuous representation and exposure of d^2/m, the term $y_i(x_{i-1} - 2x_i + x_{i-1}) + x_i(y_{i-1} - 2y_i + y_{i-1})$ is transformed to $c\nabla^2 c$ (in the concentration phase field notation). Term (B) Equation A.5 can be recognized in this expression. The enthalpy of mixing and gradient penalty terms can be recognized in addressed terms. Recognition of enthalpy of mixing is straightforward, since it corresponds to the quadratic term in the total free energy (Equation A.5). The prefactor to this quadratic term is designated Ω (Equation A.6), which is usually called the enthalpy of mixing or the regular solution parameter. The gradient penalty coefficient κ (Equation A.7) can be recognized as the prefactor to $c\nabla^2 c$, as described by Cahn and Hilliard[3]. The enthalpy of the mixing and gradient penalty does read:

$$\Omega = \frac{Nz}{N_A c_m} \Delta\varepsilon, \tag{A.6}$$

$$\kappa = \frac{Nzd^2}{2m} \Delta\varepsilon. \tag{A.7}$$

The second part of Equation A.5 is the surface integral, which represents all the contributions of the surface to the total free energy. The surface energy of the pure phases $LiFePO_4$ and $FePO_4$ in contact with electrolyte can be written as $\gamma_{LiFePO_4} = \frac{1}{2} zm(\varepsilon_{LiLi} - \varepsilon_{LiE})$ and $\gamma_{LiFePO_4} = \frac{1}{2} zm(\varepsilon_{vv} - \varepsilon_{vE})$. Thus, the expression under the integral by definition represents the surface free energy density γ (Equation 16). With the Lagrange variational principle, the Cahn-Hilliard equation (Equation 20), the chemical potential (Equation 21) and the natural boundary condition (Equation 11) are obtained from F. F represent the Euler-Lagrange functional, which defines the Lagrangian function of the system. The Lagrangian function of a system is

obtained by use of the divergence theorem ($\int_A \gamma dA = \int_V \nabla(\mathbf{n}\gamma)dV$) in order to join bulk and surface parts under one volume integral. The Lagrange function of the system therefore reads:

$$\mathscr{L} = k_b T \frac{c}{c_m} \ln\left(\frac{c}{c_m}\right) + k_b T \left(1 - \frac{c}{c_m}\right) \ln\left(1 - \frac{c}{c_m}\right) + \Omega \frac{c(c_m - c)}{c_m^2} + \frac{\kappa}{c_m}(\nabla c)^2 + \nabla(\mathbf{n}\gamma). \tag{A.8}$$

The definition of the chemical potential of the system arises from the Euler-Lagrange equation

$$\frac{\partial \mathscr{L}}{\partial c} - \nabla \frac{\partial \mathscr{L}}{\partial \nabla c} = 0 \tag{A.9}$$

which is satisfied at the minimum of the chemical potential. This condition results in the Equation 21[4]. Insertion of the obtained chemical potential into the continuity equation results in the Cahn-Hilliard Equation 20. From the Euler-Lagrange equation (Equation A.9), another identity is obtained, the natural boundary condition[5], which is written in Equation 12[6].

References

1. Murakami, T., Ono, S., Tamura, M. & Kurata, M. On the theory of surface tension of regular solution. *J. Phys. Soc. Jpn.* **6**, 309–312 (1951).
2. McQuarrie, D. *Statistical Mechanics* (Happer & Row, New York, 1976).
3. Cahn, J. W. & Hilliard, J. E. Free energy of a nonuniform system. i. interfacial free energy. *The J. chemical physics* **28**, 258–267 (1958).
4. Cahn, J. W. On spinodal decomposition. *Acta metallurgica* **9**, 795–801 (1961).
5. Sagan, H. *Introduction to the Calculus of Variations* (Courier Corporation, 1969).
6. Cogswell, D. A. & Bazant, M. Z. Theory of coherent nucleation in phase-separating nanoparticles. *Nano letters* **13**, 3036–3041 (2013).
